# Developmental Origins of Mental Health and Disorders: A DOMHaD approach to understanding, preventing and treating psychiatric disorders

**DOI:** 10.1038/s44220-025-00495-3

**Published:** 2025-09-18

**Authors:** Sonali N. Reisinger, Anthony J. Hannan

**Affiliations:** 1 Florey Institute of Neuroscience and Mental Health, University of Melbourne, Parkville, Victoria, Australia; 2 Department of Anatomy and Physiology, University of Melbourne, Parkville, Victoria, Australia

## Abstract

This Perspective outlines developmental origins of mental health and disorders (DOMHaD) as a proposed theoretical framework, building upon the concept of the developmental origins of health and disease (DOHaD). DOMHaD is supported by accumulating evidence that all major psychiatric disorders, including those with adult onset, have their origins in development, including neurodevelopmental trajectories and spatiotemporal regulation of brain-body interactions. DOMHaD incorporates our understanding that each individual’s genome, determined at conception, makes them predisposed or resilient to one or more disorders. Overlayed on these genetic factors are environmental and epigenetic factors with origins prior to conception, via parental exposures, as well as ongoing *in utero*, postnatal, childhood and adolescent exposures. These genetic and environmental factors interact over time, revealing healthy and dysfunctional developmental paths as two sides of the same complex ‘coin’. Implications of the DOMHaD conceptual framework for the understanding, prevention and treatment of psychiatric disorders will be discussed.

## 1 Introduction

### 1.1 The increasing burden of neuropsychiatric disorders

Brain health is one of the greatest challenges facing humanity in the 21^st^ century, with an enormous and growing burden of neurological and psychiatric disorders globally. Whilst specific neurological disorders (e.g. neurodegenerative diseases) have shown signs of promising therapeutic developments in recent years, most major psychiatric disorders remain inadequately understood and poorly treated. Symptomatic treatments exist for a number of these disorders, but their efficacy is often limited and side effects can be substantial. Most classes of current pharmacological treatments (e.g. antipsychotics and antidepressants) were first developed decades ago, and progress has been frustratingly slow. There is therefore an enormous remaining challenge, to develop new approaches to prevent, treat, and eventually cure, psychiatric disorders.

To this end, a systematic approach is required to understanding their etiology, including brain development (and brain-body interactions) in healthy and disordered states. This Perspective will explore the central role of developmental biology, and particularly neurodevelopment, in our understanding of psychiatric disorders. Through the lens of genetic and environmental interactions, via epigenetics and other processes, we will explore the consequences of neurodevelopmental trajectory deviations on adult brain structure and function, and dysfunction.

### 1.2 Developmental Origins of Health and Disease (DOHaD)

DOHaD is a concept first proposed by Barker and colleagues, as they attempted to understand the fetal origins of cardiovascular disease and other common disorders^1–3^. This has led to an entire field, and scientific organisations^3^, devoted to understanding developmental processes and their implications for preventative and therapeutic medicine. However, much of the focus of the DOHaD field has remained on maternal exposures and outcomes such as cardiovascular disease and metabolic disorders. Here, we shift the focus to the developmental origins of mental health and associated disorders (DOMHaD), emphasising how environmental exposures of both parents, as well as the individual, throughout sensitive periods of brain development and maturation, interact with genetics, epigenetics and other factors to substantially contribute to the development of neuropsychiatric disorders across current diagnostic categories.

### 1.3 Developmental origins of mental health and disorders (DOMHaD)

In defining DOMHaD as a discrete subclass of DOHaD, the intention is to focus attention, and future research priorities, on the fact that the major psychiatric disorders originate in development – with important implications for the development of novel approaches to prevent and treat these disorders.

The diploid genome of each individual is formed at conception from two parental haploid genomes. Genetically determined programs of embryonic and fetal brain development can be modulated by environmental exposures to the individual from conception onwards, directly or indirectly. Indirect modulation can occur via effects on epigenetic marks, which include modifications such as DNA methylation, histone modifications, and multiple classes of non-coding RNAs (ncRNAs). These are not only sensitive to environmental influences on an individual, but can reach back, if transmitted through the haploid sperm and oocyte via epigenetic inheritance, to preconceptual parental (and grand-parental, etc.) exposures and lifestyle factors. During pregnancy, the mother’s environmental exposures and lifestyle act in concert to influence innate genomic and epigenomic programs controlling intrauterine fetal ontogenesis, thus steering the individual’s developmental trajectory. In the early postnatal phase, environmental influences can have long-lasting effects on brain maturation and function due to the experience-expectant neural mechanisms at play, where external factors guide crucial steps in CNS maturation^4^. The human brain then continues to develop into early adulthood, when higher cognitive functions and social competency reach maturity after additional periods of developmental plasticity^5–7^, when the brain once again shows increased vulnerability to disturbances, with potentially dire consequences for mental health^8–11^. Indeed, a meta-analysis including >700,000 individuals has recently revealed that the peak age of onset across all classes of psychiatric disorders is only 14.5 years, with almost two-thirds of patients diagnosed before the age of 25^12^. This finding highlights the importance of brain maturation processes in both the pathophysiology and the potential for preventative measures at early timepoints for the majority of patients affected by mental illness, whether they are effected by disorders classically considered as ‘neurodevelopmental’ (e.g. autism spectrum disorder, ASD) or not^12^.

The DOMHaD framework integrates early developmental phases and maturational processes during important periods of brain plasticity and accompanying vulnerability throughout childhood, adolescence and adulthood. DOMHaD is complex and heterogeneous, and incorporates gene-environment interactions extending from transgenerational epigenetic inheritance^13–16^, through to adolescent experiences and lifestyle factors impacting adult brain health. In proposing this extension to the DOHaD concept, we aim to enrich the original framework and to call attention to its core principles in the context of mental health research. Although molecular mechanisms may overlap, DOMHaD will serve to delineate a specific theoretical framework for neuropsychiatric disorders for researchers and mental health professionals.

## 2 Critical and sensitive periods of brain development impacting susceptibility and resilience to psychiatric disorders

The concept of critical periods of brain development has its origins in early work on the effects of sensory stimulation, and deprivation, on cortical maturation and plasticity, which has led to a detailed understanding of these processes and their sensitivity to environmental stimuli^17–20^.

**Figure 1:**
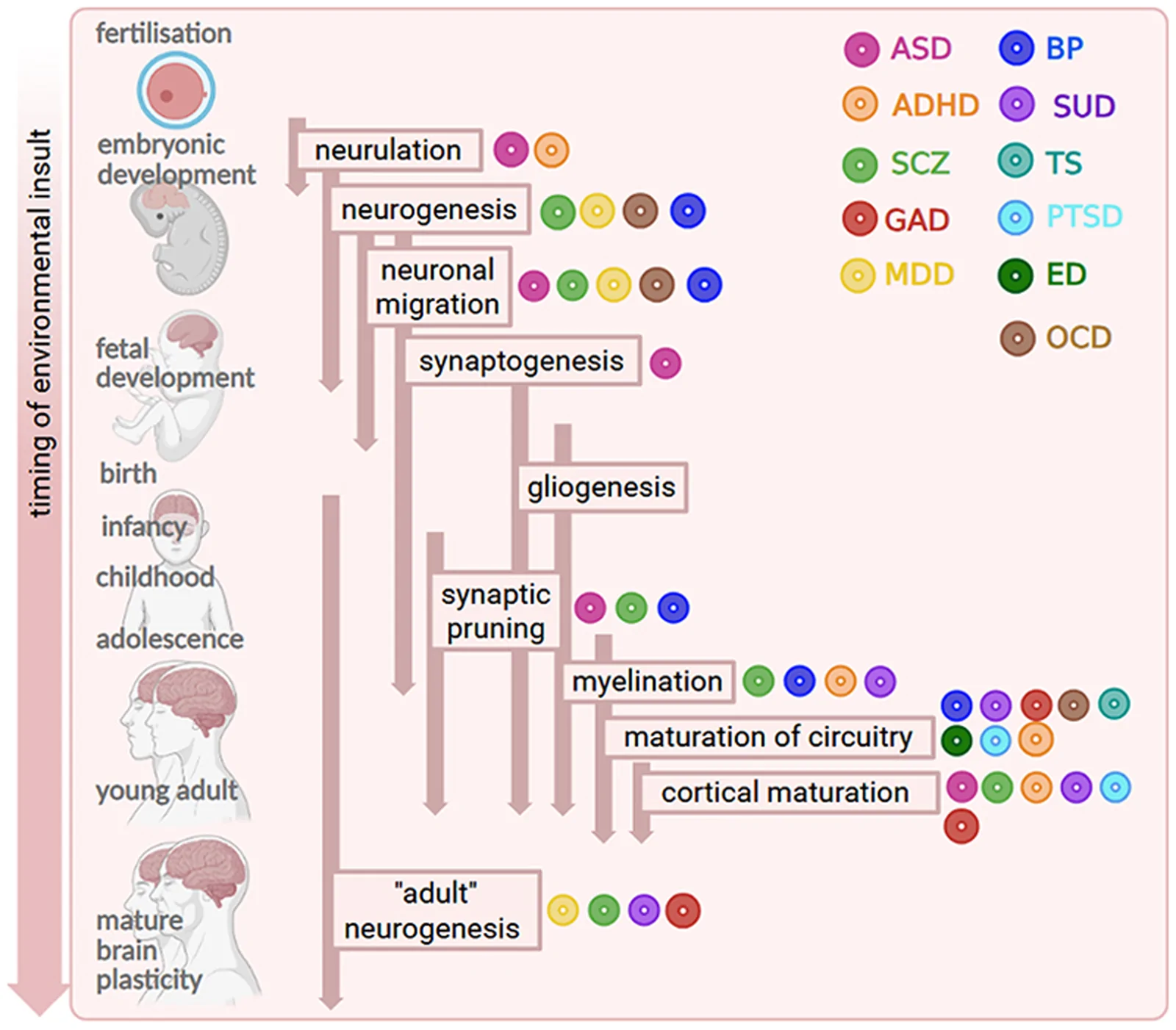
Critical periods of development impacted in the major neuropsychiatric disorders Disturbances in specific brain developmental and maturation processes, occurring from embryonic development through to adulthood, have been linked to increased individual risk for a range of neuropsychiatric disorders. These associations, mainly from human brain imaging and imaging genetics studies, highlight the fragility of human brain development, and the consequences of deviations from the optimal or typical neurodevelopmental trajectory, often via converging mechanisms. For many disorders, the role of developmental processes remains underexplored, and more research is needed to clarify the suggested neurodevelopmental mechanisms involved. Abbreviations and references: ADHD: attention-deficit hyperactivity disorder^21–27^; ASD: autism spectrum disorder^28–33^; BP: bipolar disorder^34–38^; ED: eating disorders^39^; GAD: generalised anxiety disorder^40–42^; MDD: major depressive disorder^34,42^; OCD: obsessive-compulsive disorder^34,43,44^; PTSD: post-traumatic stress disorder^45–47^; SCZ: schizophrenia^34,48–53^; SUD: substance use disorder^54–59^; TS: Tourette’s syndrome^60^. Note: High-quality reviews have been included where the body of evidence is large or complex.

In general, critical periods refer to timepoints in development reflecting crucial functional and/or structural changes in the brain, oftentimes accompanied by significant behavioural changes, which are exquisitely sensitive to external influences and insults. Therefore, these periods represent times during which the impact of environmental insults is magnified compared to less sensitive periods. This high-sensitivity state can be seen at the level of specific brain circuitry, as in the historical work by Hubel & Wiesel showing the dependence of visual cortex maturation on appropriate visual stimulus input to the eye^18^, but also extends to broader views of experiences and resulting behaviours during sensitive periods. Seminal animal research by Harlow and colleagues in the 1950s and 1960s provided some of the first experimental evidence that early life experiences and exposures strongly influence behavioural development and interpersonal relationships later in life^61,62^. By examining the effects of early infant isolation and the role of surrogate mother characteristics, they were able to demonstrate the importance of frequent intimate contact comfort for emotional development (‘cloth mother’ vs. ‘wire mother’ experiments^62^). Building upon the earlier work of Konrad Lorenz and others, who had investigated the phenomenon of imprinting between infant and mother^63^, these and following studies established the importance of early life experiences on behaviour and mental health later in life. Today, in addition to disturbed parental bonds and relationships, childhood sexual or physical abuse, and early exposure to violence or substance abuse, are considered the main types of adverse childhood experiences (ACEs), and these are now established as strong predictors of later neuropsychiatric diagnoses by several meta-analyses^64–67^. Later, adolescence is another period of enhanced sensitivity to the environment, due in part to the plasticity and maturation of the reward and motivation circuitry of the brain in this phase, which has been suggested to enhance risk taking and novelty seeking^68^. Indeed, these brain changes are typically accompanied by behavioural changes in young adults, which in turn may contribute to the development of neuropsychiatric disorders, for instance in the case of adolescent cannabis use leading to the precipitation of schizophrenia (SCZ) in predisposed individuals^69,70^. More recently, the need for a differentiation between ‘critical’ and ‘sensitive’ periods of development has been put forward, due to the observation that environmental impacts during certain periods, termed ‘critical’ periods, will have permanent and long-term consequences, while other periods appear highly sensitive to disruptions, but a recovery is possible later on – given the right environmental context (‘sensitive’ periods)^71^. This highlights the potential for early and targeted interventions to counteract certain aberrations in brain developmental processes. For instance, a number of studies over past decades have investigated the long-term impacts of orphan institutionalisation during the Ceauşescu dictatorship (1967-1989) in Romania on a range of cognitive and psychological parameters^72,73^. Researchers found that early adoptions (up to 24 months of age) into stable foster families could rescue many of the deleterious effects on cognition resulting from the neglectful environment children were exposed to in state institutions, with the length of institutionalisation being the strongest predictor of this outcome^72^. Importantly, it seems like these effects persist into adulthood, as shown by a recently published update on the longitudinal randomised controlled trial with the Romanian orphans, who are now young adults, and also extends to language skills, social functioning, rates of psychiatric diagnoses (except ADHD), and brain structural outcomes such as reduced cortical thickness^73^. Thus these findings not only illustrate the long-term and potentially severe consequences of early life deprivation and adversity, they convincingly show that when these deleterious environmental factors are recognised, it is still possible to alter the trajectory of brain development to ensure individuals can thrive later in life. Natural experiments such as the Romanian orphan studies are relatively rare, thus the comprehensive interrogation of specific sensitive periods of brain development, in terms of deleterious outcomes in the case of disruption, and the potential (and specific parameters) for rescue of negative outcomes, remain difficult to ascertain. However, through proposing DOMHaD, we seek to support researchers and mental health professionals in providing a framework integrating findings from a range of different fields of research, which converge on the neurodevelopmental origins of neuropsychiatric disorders, and which we hope will, in time, provide evidence-based and targeted treatment approaches within this framework. Since the maturation of brain areas essential for higher order cognitive processes, e.g. the prefrontal cortex, extends into adolescence and early adulthood, these late developmental processes and critical or sensitive periods are incorporated into the DOMHaD concept, rather than focusing exclusively on prenatal and perinatal processes impacting brain health^74^. Much of the gyrification of the human cortex, as well as neuronal and glial cell maturation and synaptic pruning, occur during childhood, adolescence and into adulthood, and one or several of these essential processes are thought to be disturbed in most of the major neuropsychiatric disorders (Figure 1). Overall, the importance of critical and sensitive periods in the pathophysiological processes leading to mental illness later in life has been demonstrated time and time again, thus it represents one of the fundamental pillars upon which the DOMHaD framework rests. However, DOMHaD also incorporates several other biological concepts that converge on to brain developmental processes, and which we posit may lead to the manifestation of psychiatric disorders, which will be discussed in the following sections.

## 3 Environmental influences sculpting brain maturation and function

**Figure 2:**
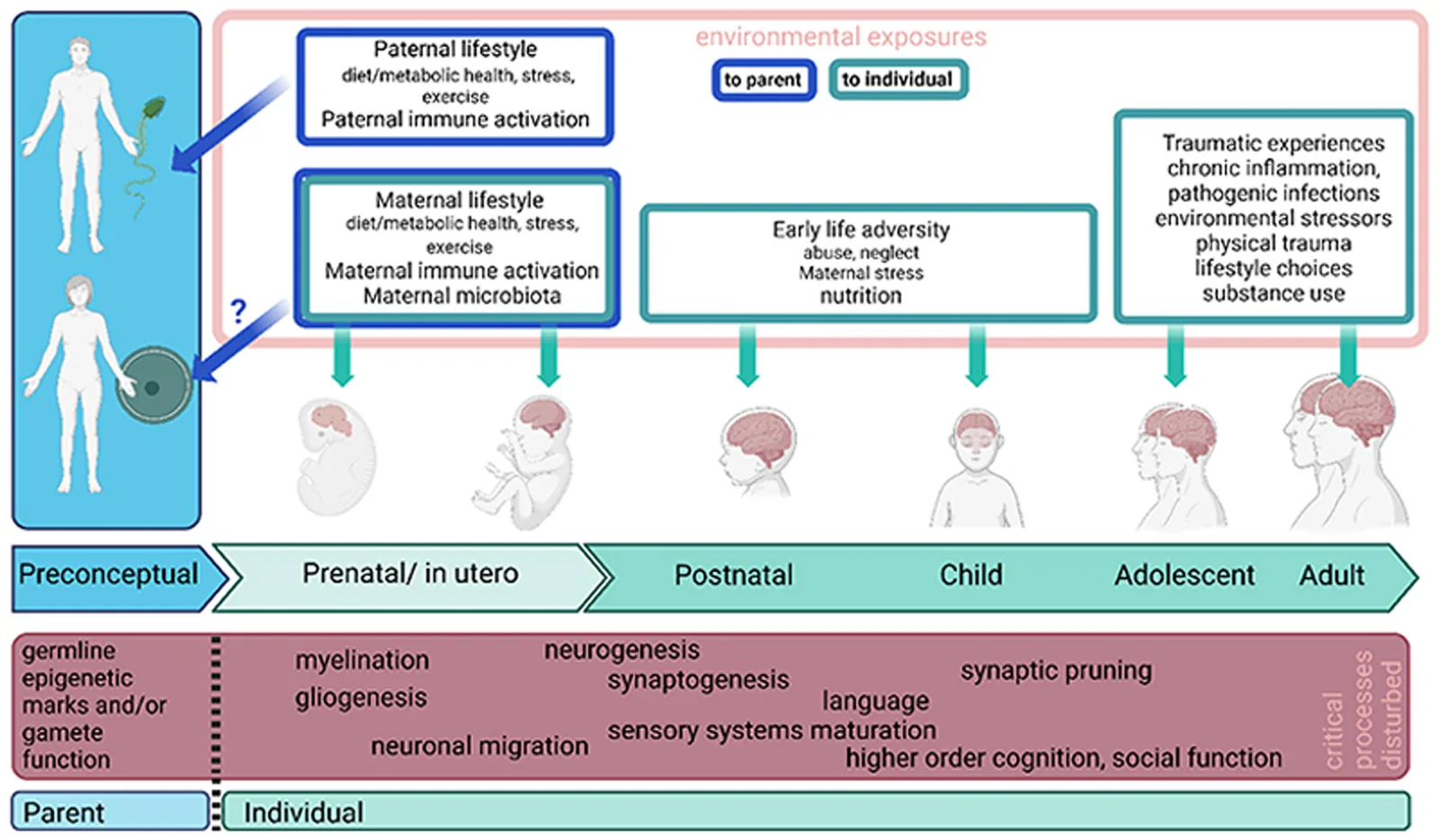
Environmental exposures to the parental generation and the individual causing disturbances in neurodevelopmental processes over time Environmental effectors may impact directly on the individual to cause disturbances to the optimal brain developmental trajectory when these occur prenatally (in utero) or postnatally. Preconceptional environmental exposures to mothers and fathers, and parental lifestyle factors, including diet and pathogenic infections, impacting on sperm and oocyte health and function prior to fertilisation, have been increasingly linked to changes in offspring (and grand-offspring) mental health status, mediated at least in part by changes in developmental processes. Consequently, the impact of environmental exposures on brain development is not restricted to the individual, but may reach back several generations.

After fertilisation, when the sperm has delivered both its haploid genome and epigenetic cargo to the haploid oocyte, a cascade of embryonic mechanisms begin to unfold. The diploid genome of the individual at conception encodes a wide range of phenotypic possibilities, interacting with environmental, epigenetic and stochastic factors throughout development. Critically, these non-genetic factors are able to impact the genetically determined developmental programs not only during embryonic and fetal development, as well as postnatal maturation processes throughout childhood, adolescence and early adulthood, but can also have significant effects even when their timing precedes conception (Figure 2). While a comprehensive overview of environmental exposures and their impact on neurodevelopment is beyond the scope of this Perspective, it is difficult to overstate the significance and diversity of such exposures, many of which are unavoidable facts of life. Stress or adversity, immune activation, pollution, exposure to toxicants such as pesticides or PFAS (per- and polyfluoroalkyl substances, found in many consumer products) have been shown to have mostly deleterious effects to the developing brain of the individual, or the individual’s progeny (reviewed in^75–77^), though there are not many well-controlled and -powered studies in humans. Dosage and timing of exposures appear to play a significant role, highlighting once again the existence of sensitive periods. New environmental concerns are constantly emerging, where effects on brain development are still largely unclear, for example those of micro- and nanoplastics penetrating our tissues, or the effects of extreme temperatures accompanying the current climate crisis. As scientists uncover the specific effects of particular exposures on neurodevelopment, it will be crucial to identify common pathways and molecular mechanisms which the environment is able to influence, at specific timepoints, to gain a holistic understanding of the brain’s vulnerability over time, and to be able to develop strategies protect the developing brain (and gametes, in the case of preconceptional exposures) against the negative consequences of existing in an evolving environment.

### 3.1 Pre-conceptional impacts on the developing brain

When considering pre-conceptional exposures to the parental generation in relation to offspring brain health, it is important to recognise that most environmental exposures exist on a spectrum of intensity and/or severity. Additionally, offspring neurodevelopmental outcomes may differ significantly depending on the timing of the exposure relative to conception, highlighting the need to study environmental exposures using a variety of protocols.

Increasing evidence from animal studies suggests that the epigenetic cargo (e.g. non-coding RNA) delivered by spermatozoa to the oocyte upon fertilisation is significantly altered by a variety of environmental exposures including diet^78,79^, stress^14,15,80^, environmental toxicants^81^, pathogenic infection^16,82^ and physical exercise^83^. Furthermore, increased psychiatric diagnoses (in human studies^84–88^) or behaviours relevant to neuropsychiatric disorders (in animal studies^14–16,80,82^) in subsequent generations have been reported after several of these environmental insults. Instances of inter- and transgenerational trauma have been some of the most widely reported cases of heritable preconceptional exposures, including several studies in Holocaust survivors and their children, who experienced significantly higher rates of PTSD^85,87^ and showed differences in several stress response parameters^86,89^. In general, the sample sizes in these studies were small and self-limited, and while other studies have partially replicated the findings in other populations, such as those who had survived the genocide against the Tutsi people of Rwanda in the 1990s^90–92^, these types of retrospective studies are inherently difficult and few are able to reliably distinguish the indirect impacts of paternal trauma on children (e.g. via altered parenting behaviour) from instances of true intergenerational inheritance. Also, few have focussed specifically on the impact of parental trauma on brain development. Of those that have, several use third-trimester fetal heart rate variability (FHRV) as a proxy index for neurodevelopment, as this is intimately linked to the development of the parasympathetic nervous system^93,94^, which has the advantage of being independent of parenting behaviour. One study found no direct link between childhood maltreatment and alterations in FHRV, including maternal sleep disturbances during gestation as a latent variable resulted in a significant association, suggesting that some effects of parental trauma on neurodevelopment may be indirect^93^. However, a separate study looking at the effects of childhood trauma in a population of mothers diagnosed with bipolar disorder found that there was a significant effect of maternal trauma on FHRV, as well as gestational age at birth and birth weight^94^, both of which are also highly relevant risk factors for neurodevelopmental and neuropsychiatric disorders^95–99^. These may represent confounding factors, and it is unclear whether these findings are generalisable, due to small sample sizes, however they highlight the utility of using fetal physiological measures. Longitudinal studies with follow-ups are required to confirm the relevance of FHRV to neuropsychiatric diagnoses.

Mechanistically, the transmission of epigenetic information (long and short ncRNAs, possibly DNA methylation and histone modification marks), is thought to form the basis of inter- and transgenerational epigenetic inheritance via the paternal line^100^. The ability of certain epigenetic marks to evade the epigenetic reprogramming mechanisms in early mammalian embryonic development, is still an area of intense research. Genomic imprinting, where one allele of a gene is silenced completely depending on the parent-of-origin, is the most well-characterised of these mechanisms, and is thought to occur through the maintenance of DNA methylation via a number of different molecular mechanisms (reviewed elsewhere^101^). More recently, some transposable elements have also been shown to escape waves of demethylation^102–104^, and histone-based inheritance has also been reported^105,106^. The precise role of sperm epigenetic cargo in early embryonic development is being uncovered only recently, using advances in next-generation sequencing methods that allow the study of transcriptional profiles in early embryos at the single cell level^107^. In mice, paternally-derived RNAs influence implantation and regulate the expression of maternal miRNAs in the zygote, with important consequences for downstream fetal development^107–110^. These types of developmental changes may significantly impact later CNS development via modified brain maturation trajectories. On the other hand, several offspring health effects have been linked to aberrant placental development^111–113^, although none of these studies have established a causal link with brain developmental abnormalities or examined adult behaviour to date. Intriguingly, fathers are thought to contribute factors essential to placenta formation and function^114–116^, and the placenta plays a key role in brain development. Functional changes in the placenta have been associated with aberrant brain maturation, behaviour, as well as mental health issues later in life^117–119^, thus could represent a plausible mediating mechanism for paternal epigenetic inheritance.

On the other hand, mechanisms underlying pre-conceptional environmental exposures in mothers on offspring development are less well-studied. This may in part relate to the perceived stasis in female germ cells throughout an individual’s life, as all oocytes are generated prenatally, while spermatozoa are produced continuously starting in puberty^120^. However, recent evidence from primate and rodent studies suggests that oocytes are also susceptible to environmental exposures such as dietary sugar or chemotherapy drug toxicity, with notable functional deficiencies in oocyte maturation and embryo development demonstrated *in vitro* and *in vivo*^121^. In humans, lifestyle factors such as maternal obesity are thought to influence a range of oocyte parameters, however deleterious effects on offspring health resulting from preconceptional lifestyle choices are difficult to disentangle from those effects impacting on development during gestation. For maternal obesity, human epidemiological studies indicate that a higher maternal pre-pregnancy BMI predicts lower cognitive performance in offspring^122^, issues with emotional regulation in childhood and adolescence^123^, and higher rates of autism spectrum disorders (ASD)^124^, attention-deficit/hyperactivity disorder (ADHD)^125^, cerebral palsy^126^ and SCZ^127^ later in life (for a comprehensive review, see ^128^). The current literature on pre-conceptional micronutrient deficiencies (e.g. folic acid to prevent neural tube defects^129^) and other pre-conceptional maternal factors (e.g. reproductive tract infections^130^, medications^131^) supports the notion that maternal influences on offspring health also start pre-conceptionally, although a direct link to increases in neuropsychiatric diagnoses in the offspring have not yet been demonstrated. Transgenerational propagation of oocyte epigenetic alterations after exposure to the chemotherapy drug cyclophosphamide has been shown in mice, providing the first evidence for maternal-line epigenetic inheritance, although the authors did not investigate offspring behaviour or brain development^132^. This type of inheritance may also be highly relevant when considering the outcomes of assisted reproductive technologies, as it has been suggested that oocyte ‘vitrification’ is associated with a number of epigenetic modifications, although human and animal studies have reported mixed results on this topic^133–136^. Potential effects on offspring neurodevelopment and later psychiatric diagnoses are still up for debate and require further research^137–139^.

### 3.2 In utero environmental factors and exposures modulating brain development

As mentioned above, *in utero* development, including brain development, has major impacts on postnatal health and susceptibility (or resilience) to a wide range of disorders (Box 1). For example, there is large body of evidence from both human and animal studies supporting the notion that maternal infection, leading to maternal immune activation (MIA) during specific stages during pregnancy, can alter fetal brain development in ways that increase later susceptibility to specific neurodevelopmental and psychiatric disorders, including ASD and SCZ^140–145^. The evidence for increased incidence of mood disorders, including major depressive disorder (MDD) and bipolar disorder (BP), is slightly less robust and appears to relate to infections occurring at distinct time points of gestation, supporting the notion that different neurodevelopmental processes may be impacted in these groups of disorders^146–151^. This weaker level of evidence could also relate to the relatively stronger influence of additional postnatal environmental factors on the risk to develop affective disorders, as opposed to ASD and SCZ where early development is thought to play a more central role^152–154^.

In addition to maternal infection, many other factors impacting on maternal health and wellbeing may also significantly influence the developing fetus and brain. Psychological stress (Box 1) and psychiatric diagnoses such as anxiety and depression during pregnancy have been linked to brain developmental changes in offspring, with mid-gestation emerging as a particularly sensitive period^155–160^. Studies looking at exposure to toxicants during pregnancy, including lead^161^, fine particulate matter (atmospheric pollution from fossil fuel combustion and other sources)^162,163^, and endocrine disruptors (e.g. phthalates)^164–166^ suggest deleterious impacts on brain structure, microstructure, and cognition. However, there is an urgent need for large prospective studies with longitudinal designs, and the additive impact of different types of exposures needs to be specifically investigated, considering the ubiquitous presence of various toxicants in our environment and our bodies.

### 3.3 Postnatal environmental impacts on brain development and maturation

While a number of environmental impacts such as specific sensory stimuli or stressors can cause decanalisation of brain maturation trajectories when occurring during critical or sensitive periods^214^, prolonged exposure to particular environmental conditions or lifestyle factors can also significantly alter brain development and contribute to pathophysiological processes culminating in a mental illness diagnosis.

As a salient example of this association, accumulating evidence suggests that there is a link between certain nutritional factors and neuropsychiatric diagnoses. In particular, both a shift in dietary habits towards unhealthy combinations of processed foods, high-sugar and/or high-fat meals, and a reduction in vegetable intake^215^; but also a deficiency in specific essential nutrients (e.g. fatty acids^216^) have been highlighted as potentially important contributors to the epidemic increase in specific mental illnesses across the globe. Regarding the case of fatty acid intake, which includes omega-3 (ω-3 PUFA) and omega-6 (ω-6 PUFA) polyunsaturated fatty acids, among others, this has been linked to psychiatric disorders canonically considered to originate in neurodevelopment (ASD, ADHD, SCZ), but also to mood disorders^216^. PUFAs are essential constituents of neuronal cell membranes, therefore it is perhaps unsurprising that reduced availability of these molecules may have deleterious consequences on brain health. However, they may be particularly crucial during specific brain developmental stages, particularly a form of ω-3 PUFA called docohexaenoic acid (DHA), as the formation of neurons and glia in the late stages of pregnancy and early postnatal life poses increased requirements for these nutrients^217^. DHA is present in breast milk, in quantities directly related to maternal DHA intake, however not all milk formulas contain this essential PUFA, and this deficiency has been suggested to underlie long-term differences in neurodevelopmental and cognitive outcomes observed between breastfed and formula-fed children^218–220^. Adequate DHA intake is now considered to be essential prenatally as well as in the early postnatal phase in particular for retinal maturation and early brain growth, and in later stages of childhood it has been shown to improve cognitive outcomes in some studies^221,222^, although others have failed to report positive effects^223^. DHA has been shown to play important roles in neurogenesis, myelination, neuroplasticity processes including neurite outgrowth and long-term potentiation, pre- and postsynaptic density organisation^224–227^. These are all processes related to brain maturation and plasticity, or maintenance of brain health, and when disturbed have been associated with the development of neuropsychiatric disorders (see Figure 1), thus one might speculate that the relevance of PUFAs in mental health is due in large part to their role in these essential developmental processes.

## 4 Mechanisms underlying deviations from typical brain development

### 4.1 Genetics

#### 4.1.1 Genetic factors leading to altered brain developmental trajectories

Across psychiatric and neurological disorders, while genetic contributions can be substantial, few single genes or polymorphisms have convincingly and consistently been shown to have strong risk-conferring effects. Indeed, as for many complex diseases, most gene candidates which have been associated with specific illnesses tend to create a predisposition rather than being highly penetrant risk factors, and further environmental impacts are thought to be required for disease initiation.

Nevertheless, research in the past several decades has uncovered a number of genes that contribute to increasing the risk for CNS disorders. Crucially, a substantial proportion of these code for proteins which are directly involved in neurodevelopmental processes, providing a further anchoring point for the DOMHaD perspective neuropsychiatric disorders.

**Figure 3:**
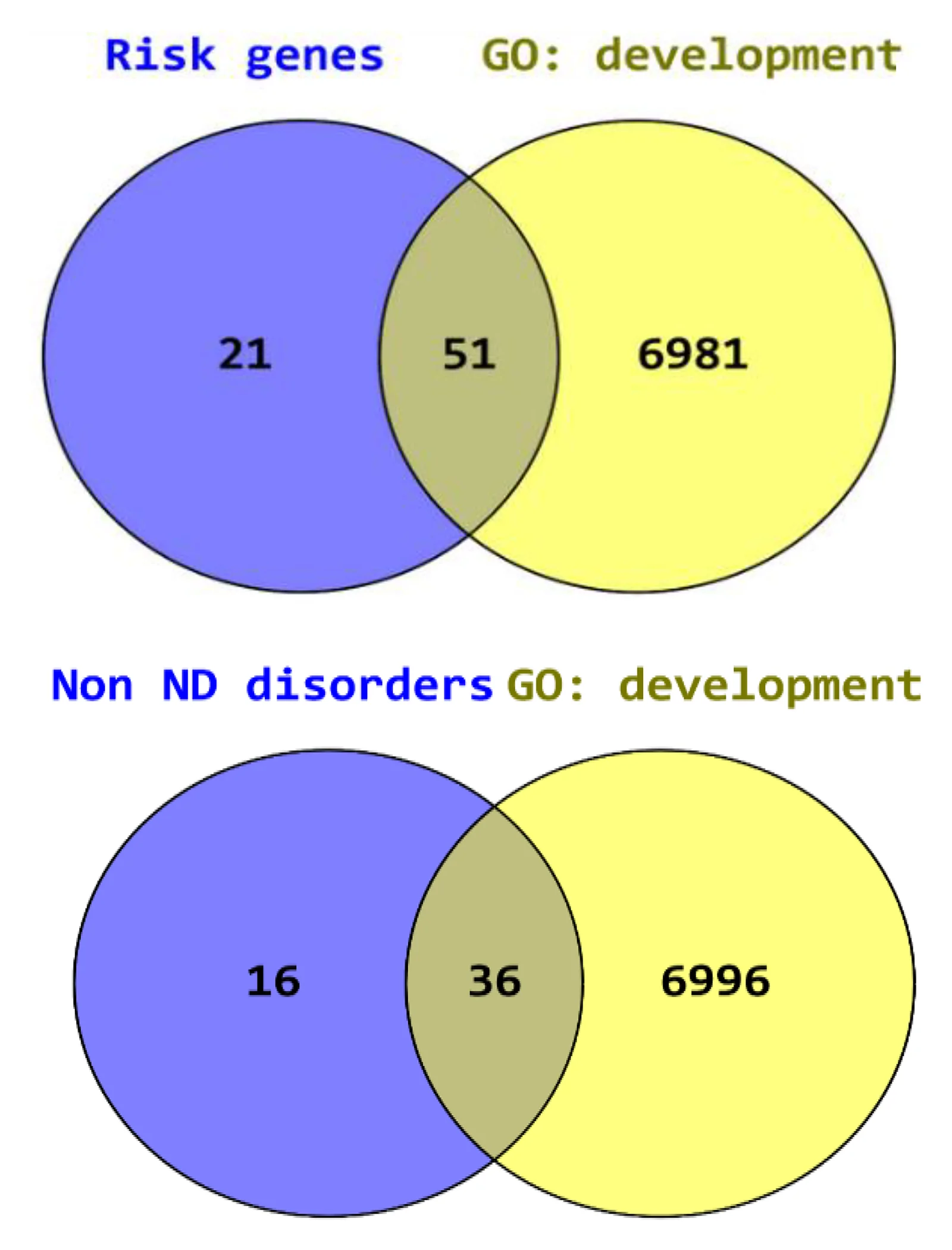
Risk genes for major neuropsychiatric disorders vs. gene ontology set “development” *Top panel*: Overlap between risk genes for the major neuropsychiatric disorders (top 10 for each disorder according to DisGeNet database) and genes included in the gene ontology annotation list for developmental processes. *Lower panel*: Overlap between risk genes for the neuropsychiatric disorders generally considered to not be related to neurodevelopment (top 10 for each disorder according to DisGeNet databae) and genes included in the gene ontology annotation list for developmental processes. Disorders included in search: ‘neurodevelopmental disorders’ -schizophrenia, autism spectrum disorder, attention deficit hyperactivity disorder; ‘non neurodevelopmental disorders’: major depressive disorder, bipolar disorder, anxiety disorders, obsessive-compulsive disorder, substance use disorder, Tourette’s syndrome.

For instance, the DISC1 (disrupted in schizophrenia 1) gene was considered a risk gene for SCZ and other major mental illnesses, after it was first identified in a large Scottish pedigree study^228,229^. More recent, larger studies have reported predominantly negative results in terms of its risk-conferring role^230–233^. An alternative approach takes into account the entire network of proteins interacting with DISC1 (or ‘interactome’), in acknowledgment of the highly polygenic nature of SCZ and other neuropsychiatric disorders^234–237^. One study has applied this approach to the Psychiatric Genomics Consortium GWAS dataset, arguably the only adequately powered GWAS of psychiatric patients to date^230,237,238^. In this study, significant associations were found between several common variants of different genes in the DISC1 interactome and SCZ, via several complementary analysis methods, providing highly convincing evidence^237^. Intriguingly, DISC1 and its interactome has been shown to play integral roles in both fetal and postnatal brain development, including the control of cortical maturation, by several lines of evidence^239–242^, and its association with a cluster of mental illnesses further supports a pathophysiological relevance of these processes beyond the ‘classical’ neurodevelopmental disorders (mainly ASD, ADHD and SCZ). When examining other risk genes, this pattern is repeated: Of the top ten risk genes for each of the major neuropsychiatric disorders (according to the database DisGeNet^243^), a majority of ~70% are part of the gene ontology category ‘development’, whether one includes ASD/ADHD/SCZ as the ‘classical’ neurodevelopmental disorders (Figure 3, top panel), or only those disorders that are traditionally considered less dependent on neurodevelopment (MDD; BP; GAD; obsessive-compulsive disorder – OCD; substance use disorder – SUD; Tourette’s syndrome – TS; Figure 2, lower panel). While this is not proof of causal involvement, this certainly suggests that gene networks related to neurodevelopment are likely important across all the major mental illnesses, at least based on the currently known genetic associations. This assertion is also backed by imaging genetics findings in recent years. Studies of infant^53^, adolescent, and adult brains^244,245^ have revealed that combinations of common genetic variants of psychiatric risk genes are able to predict differences in brain structure, providing an important link between genetic variants and brain structure as endophenotypes highly relevant to neurodevelopmental outcomes.

It is however important to recognise that not all developmental risk genes necessarily exert their effects within the brain. This was demonstrated in a compelling set of studies showing that schizophrenia-associated genes are highly expressed in the placenta during development, that their expression is dynamically regulated, and that these expression patterns interact with early life adversity in a sex-biased manner to influence schizophrenia risk^246,247^. This highlights the importance of tissue-specific functional genomics studies, including in extra-neural tissues, in the elucidation of molecular mechanisms contributing to neuropsychiatric risk, and potential resilience mechanisms.

Therefore, while genetic risk factors in mental health are individually not highly deterministic, polymorphisms in many of the relevant genes actually predispose the individual to a kind of neurodevelopmental ‘instability’, which, when coupled with specific environmental exposures, are more likely to result in deviations from ‘normal’ neurodevelopmental trajectories (i.e. decanalisation^214^) than in individuals not carrying these risk factors. Alterations in brain maturation processes, in turn, lead to structural and/or functional abnormalities which mechanistically underlie the observed symptom complexes, and these processes are important not only in SCZ, ASD and ADHD where neurodevelopmental processes have long been implicated, but across the major neuropsychiatric diagnoses.

#### 4.1.2 Gene-environment interactions shaping neurodevelopmental processes

Outside of the genetic control of brain development, which undoubtedly is a key mechanism relevant here, gene-environment (GxE) interactions also play a role in several ways. The observation that individuals exposed to similar detrimental environmental stressors (such as prenatal adversity) do not usually show the same level of susceptibility to a particular disorder later in life, and on the other hand, that risk-associated genes virtually never show complete penetrance in psychiatric genetics, had first been made in the 1960s in the context of schizophrenia^248,249^. However, the methodological challenge of quantifying and systematically studying gene-environment interactions has meant that significant advances in this space have only been made in the last few years. Indeed, the recent development of new statistical approaches that are able to model complex higher-level interactions with sufficient power has led to the confirmation of GxE interactions as a crucial framework for understanding psychiatric risk. In the context of ADHD, a 2021 study used a new statistical approach termed LEGIT (Latent Environmental and Genetic Interaction Tool)^250^ to study the interaction between prenatal adversity, early postnatal environment (including maternal care) and gene variants for several genes related to the dopaminergic machinery, which is thought to be heavily involved in ADHD, and the combined impact on attentional function in early childhood^251^. While the authors were not able to validate a significant three-way interaction between these factors across different samples, potentially pointing towards a small effect size, they found several robust interactions between prenatal adversity and dopaminergic genotype, prenatal adversity and early maternal behaviour, as well as dopaminergic genotype and early maternal behaviour, impacting on attentional outcomes in children^251^. Another crucial aspect of GxE interactions has been termed ‘genetic nurture’^252^: Parental genotypes, even when not transmitted to offspring, can shape development through their influence on the environment the child is exposed to, including parenting styles, but also more distal factors influenced by parental genetics, such as employment and income. Importantly, the indirect effect of genetic nurture on individual disease risk (neuropsychiatric or otherwise) had been completely disregarded in GWAS studies, although it may have amplified the significance of genetic associations in many cases^252^, a fact that may help explain low reproducibility rates. When examining educational attainment in association with polygenic risk scores, as well as parental genotypes (i.e. including information about the non-transmitted allele and the transmitted alleles at the specified loci), studies have found that genetic nurture contributes significantly to the outcome, although this type of analyses does not enable the identification of specific environmental factors^252,253^. Among neuropsychiatric disorders, ADHD has been the most studied in terms of genetic nurture contributions to disorder risk and associated endophenotypes, but these have predominantly found little to no contribution to ADHD risk or ADHD-associated traits^254–258^. In combination with the findings discussed above, i.e. significant interactions between the postnatal environment and genetic ADHD risk, the lack of indirect genetic effects in the case of ADHD suggests that the relevant differences in maternal care are not mainly driven by parental genetics. While highly complex, the development of new statistical methods, and the increasing use of family-based designs where parent-child trios (or dyads) are included in the study populations, will be extremely valuable in trying to disentangle the contributions to psychiatric risk and resilience in the future. Applying these methods across neuropsychiatric diagnoses, neuropsychiatric endophenotypes and brain structural studies will begin to illuminate the complex interplay of gene–environment interactions, offering insights not only into the underlying pathophysiological mechanisms, but also into how such effects might be modulated through targeted positive environmental interventions.

### 4.2 Epigenetic aspects of DOMHaD

#### 4.2.1 Environmental control of epigenetic mechanisms in neurodevelopment

As outlined above, environmental factors impacting on previous generations, as well as throughout the development and lifetime of an individual, play a central role in the determination of brain developmental trajectories, profoundly influencing brain function and behaviour. Epigenetic mechanisms are key mediators of these GxE interactions, as they can be altered and modulated without changing genetic information. As such, epigenetic factors, which include DNA methylation, histone modifications, and non-coding RNAs, serve as a molecular interface between environmental exposures and sustained alterations in gene expression^259^. While a comprehensive overview of epigenetic mechanisms in the pathophysiology of psychiatric disorders is beyond the scope of this Perspective, these molecular effectors of GxE interactions represent a crucial mechanism that is central to the DOMHaD theory: The alteration of epigenetic marks at specific genetic loci can not only influence expression levels in the adult and mature brain, but may have particularly far-reaching consequences when these alterations lead to variations in expression during developmental processes. In a seminal study, Weaver et al. first showed in rodents that maternal care behaviour influences epigenetic markers in the offspring^260^. This not only impacted gene expression of the glucocorticoid receptor in the hippocampus of progeny, but also resulted in an altered stress response, illustrating how early life environmental exposures can have long lasting impacts on behaviour and physiology and initiating the field of environmental epigenetics^260^. Indeed, a meta-analysis found that genes involved in neural development, as well as stress responsivity and synaptic function, showed differential epigenetic signatures across several neuropsychiatric disorders, including MDD, GAD and SCZ, highlighting the potential involvement of epigenetic regulation in neural development and stress responsivity pathways as shared mechanism among mental illnesses^261^. A key example of illustrating the importance of epigenetic regulation in psychiatric disorders relates to brain-derived neurotrophic factor (BDNF), hypermethylation of which has been associated with a higher risk of MDD, and interestingly, these epigenetic marks (and BDNF expression) appear sensitive to antidepressant treatment^262–266^. BDNF plays crucial roles in neurodevelopment, particularly in the control of synaptic plasticity, neuroprotection and neurogenesis. While it remains difficult to disentangle the acute effects of reduced BDNF expression from the long-term consequences on brain developmental processes, especially in humans, animal and human studies suggest that epigenetic regulation of BDNF is sensitive to negative environmental insults in early life, indicating that BDNF methylation may constitute a biomarker for early-life adversity and thus highly relevant for the diagnosis of several psychiatric disorders^267,268^. On the other hand, numerous studies have demonstrated that DNA methylation patterns and other epigenetic marks change significantly from birth through adolescence, influenced by both intrinsic biological programming and environmental exposures^269–272^, highlighting the instability of early-life epigenetic signatures and their limited predictive power without longitudinal data. Also, the timing of environmental exposures and epigenetic measurement can affect observed associations, with certain epigenetic effects emerging only within specific developmental windows^273^. These findings underscore that while epigenetic marks (at the BDNF locus and elsewhere) may serve as a biomarker of early-life adversity or risk, studies must account for developmental timing, tissue specificity, as well as polyepigenicity – the cumulative interaction of epigenetic mechanisms across the genome^274^. Thus, epigenetic processes may constitute key mechanisms by which the developing brain receives input, particularly during sensitive developmental periods, but this process is highly dynamic over time and across tissues.

#### 4.2.2 Genetic control of epigenetic mechanisms in neurodevelopment

Epigenetic mechanisms appear to be uniquely malleable in response to environment exposures and experience, and can modulate gene expression, however epigenetic programming is also strongly determined by an individual’s genetics^275^. Quantitative trait loci (QTL) have been used in genetics to designate genomic regions which are associated with a quantitative outcome, and this concept has been applied to the association between certain DNA regions and variations in DNA methylation sites (mQTL), chromatin accessibility (caQTL), histone modifications (hQTL), or non-coding RNA expression (miRNA eQTL, lncRNA eQTL, circRNA eQTL). These xQTLs can control epigenetic marks and mechanisms in cis (at the same gene) or in trans (at a different gene). In the case of SCZ, integrated analysis of genomic and epigenomic datasets revealed that a substantial number of genetic variants associated with SCZ co-localised with differential DNA methylation patterns, suggesting that certain risk loci may exert their risk-conferring effects through modulation of epigenetic mechanisms^276^. More recent multi-omic studies at the single-cell level support this, showing that risk variants for SCZ, MDD, BD, and ADHD are consistently associated with differential chromatin accessibility^48^, DNA methylation^48^, and histone modifications^277,278^ at neuronal and glial enhancers, promoters, and chromatin loop anchors (connecting distal regulatory elements).

Interestingly, some xQTLs appear to be active only during specific brain-developmental windows, as shown by a comprehensive genome-wide analysis of mQTLs in fetal brain samples, which revealed more than 16,000 mQTLs^279^. These mQTLs also substantially overlapped with brain transcriptional expression QTLs (eQTLs), linking these genetic loci to the control of specific DNA methylation, and of transcription, an association that is further strengthened by their enrichment at regulatory domains such as transcription factor binding sites^279^. Building on this work, another study examined fetal brains at similar developmental stages by using cell-type resolved chromatin accessibility and 3D chromatin interaction mapping to identify the enhancers and regulatory loops active in neural progenitors and early neurons^280^. This approach revealed that numerous risk variants for SCZ, MDD, and ADHD were enriched in these neurodevelopmental regulatory elements ^280^. Heffel *et al*. performed an analysis of cortical and hippocampal tissue from fetal, paediatric and adult postmortem brains and found that chromatin architecture is progressively remodeled throughout development, with SCZ risk variants enriched in the loop anchors defining gene regulatory domains of mature neurons, suggesting that risk mechanisms established during early neurodevelopment may be further consolidated during postnatal neuronal maturation^48^ Collectively, these findings implicate the disruption of early gene regulatory programs as a key mechanism in the emergence of psychiatric vulnerability, and providing further supporting evidence for the DOMHaD framework. Expanding investigations across later developmental timepoints, different brain regions, and additional psychiatric phenotypes will be critical to fully elucidate these regulatory mechanisms.

### 4.3 The microbiota-gut-brain axis and brain development

Brain-body interactions throughout development are increasingly being recognised as key determinants of adult brain structure and function. Thus, a range of environmental factors which act initially through the periphery (e.g. diet and exercise) have major impacts on neurodevelopment via brain-body interactions. However, organisms residing within our bodies also may powerfully modulate brain mechanisms including development. The gut microbiota, the trillions of microbes (mainly bacteria) which inhabit the gastrointestinal tract, are increasingly being recognised as influencing brain development, structure, function and dysfunction. An infant’s gut is seeded with the mother’s gut and vaginal microbiota during natural birth, and neonatal gut microbiota populations are modulated by maternal skin and breast milk factors. *In utero*, the fetal gut is thought to be sterile – or nearly so^281,282^. There is evidence of gut microbiota transfer from mother to fetus during gestation, as maternal gut microbes have been detected in umbilical cord blood, placenta, fetal tissues, amniotic fluid and meconium, although the mechanisms by which this type of transfer might occur (or if indeed it does robustly occur at all) are yet to be fully elucidated^281,283–285^. However, the bacterial cell wall constituent peptidoglycan (PGN), which can be released by a range of maternal gut microbiota, has been shown to cross the placenta and into the fetal brain in mice^286^. This was also associated with increases in neuronal proliferation, transcriptional changes, and later cognitive and social behavioural alterations^286,287^. Another study recently showed that maternal microbiome depletion, in germ-free and antibiotic-treated mice, had negative effects on placenta growth and vascularisation via short-chain fatty acids (SCFAs; gut microbiota metabolites)^288^, as well as reduced axon growth in thalamocortical projections of the fetal brain^289^. Collectively, these studies suggest several potential direct or indirect influences of maternal gut microbiota on neurodevelopmental outcomes during gestation. In the postnatal phase, abnormal microglial maturation was reported in association with gut microbiome depletion, and this effect was rescued by SCFA supplementation^290^. Other studies in rodent models have shown that modulation of brain processes by the gut microbial population can also occur via the vagus nerve^291,292^, circulating metabolites^293^ (produced by microbiota) or cytokines^294^ (produced by the intestinal mucosa), microglial activity and brain ncRNAs^295^. Whether these mechanisms are causally involved in regulating neurodevelopment, and may therefore be linked to neuropsychiatric risk, is an area of active research. However, studies based on germ-free or antibiotic-treated rodent models represent extreme cases of gut microbiota depletion. Germ-free mice have many other abnormalities that can also affect neurodevelopment, such as a compromised blood-brain barrier^296^. While illustrating the power of the microbiota-gut-brain axis, high-quality human studies are urgently needed to shed light on whether more subtle shifts seen in typical human microbiota variation also significantly impact neurodevelopment, the precise role of specific microbial species, and the existence of sensitive periods.

Babies delivered via caesarean birth (C-section; an increasingly common procedure in developed countries in recent decades) have a different gut microbiota composition from those delivered vaginally^297^. Supporting the notion that microbiota play a key role in neurodevelopment, there have been numerous reports of significant associations between C-section delivery and early brain developmental abnormalities, and a meta-analysis concluded that C-sections are indeed significantly associated with increased incidence of ASD and ADHD in individuals^298^. However, analyses of delivery mode are often confounded by maternal or fetal complications, such as pre-eclampsia^299^, which are independently linked to neurodevelopmental risk. Additionally, while long-term health effects of C-section delivery appear to persist, evidence shows that differences in infant gut microbiota composition may disappear relatively quickly^300^, raising questions about alternative mechanisms. Regardless of how the gut of infants is initially colonised, microbiota may play a crucial role in brain maturation processes. Microbiota influence gastrointestinal tract development, which in turn determines the ability of the gut to extract energy and macronutrients, required for ideal neurodevelopment and establishing the microbiota-gut-brain-axis as a crucial bidirectional regulatory pathway^301–303^. First studies have recently emerged examining the infant gut microbiome and neurodevelopmental outcomes such as cognition scores, reporting a positive association between indices of gut microbiota diversity and neurodevelopmental measures^304,305^, as well as behavioural problems^306^. Nevertheless, studies in the field are heterogeneous, and sample sizes generally very low (<50 on average^307^). A recent study involving >1700 individuals of the Generation R cohort in the Netherlands found no association between gut microbiota profiles and parent-reported childhood mental health problems at 10 years old^308^. Similarly, studies investigating the link between brain structure, or brain connectivity, and the gut microbiota have reported mixed results, are heterogeneous and often underpowered^309–311^. Further research, ideally with longitudinal study designs, large cohorts, and multi-omic datasets, will be needed to provide a definitive answer regarding the role of gut microbiota in the regulation of brain development.

## 5 The DOMHaD approach to treating and preventing mental health disorders

Attempting to understand the mechanisms involved in DOMHaD may appear to be a daunting challenge. Nevertheless, we suggest that the reward of such an undertaking will be immense. Exploiting the environmental sensitivity and plasticity of the maturing brain to counteract or prevent negative effects by exerting positive influences during the plastic and sensitive periods (via environmental enrichment, exercise, nutritional interventions, psychosocial support targeted to specific periods and/or particularly at-risk individuals, including pre-conceptional coaching for prospective parents - to name a few). Identifying ‘neurodevelopmentally at-risk’ parents and their (by extension) at-risk babies, by evaluating inter- and transgenerational, environmental and genetic risk factors systematically in individuals, may in future be possible if research undertaken within the DOMHaD framework succeeds in elucidating which preconceptional, *in utero* and lifestyle factors are the most important in determining an individual’s mental health. Pre-conception, prenatal, and postnatal screening carry ethical risks including pathologising certain populations, parental anxiety, or false positives, however identifying windows of vulnerability to leverage these for preventative strategies should be a priority. New and improved frameworks to guide individualised risk prediction in clinical practice will be necessary to address concerns, however, as proposed by the EU-based FAMILY consortium^330^. We anticipate that promising therapeutic approaches will emerge from preconceptional counselling, where polygenic risk profiles, epigenetic profiling, or modifiable environmental or lifestyle risk factors could be addressed in prospective parents, regardless of sex or gender identity. We suggest that by expanding research into paternal contributions, the disproportionate responsibility placed on mothers for the health of offspring may be lightened considerably.

Targeted preventative and therapeutic approaches may address aberrant mechanisms at the timepoints most likely to yield responses, which may not only increase efficacy, but may also facilitate the move away from chronic, often life-long, pharmacological treatment plans with burdensome side effects, towards highly focal interventions – applied strategically during the most plastic periods of neurodevelopment – for mental illnesses. The current apparent ‘irreversibility’ of brain alterations seen in psychiatric disorders arising in neurodevelopment is reflected in the limited efficacy of approved treatments. Therefore, targeting the relevant pathogenic processes at early timepoints to prevent the aberrant maturation promises to yield far superior outcomes, with respect to both prevention and treatment. On the other hand, any pharmacological interventions *in utero* or early childhood would require robust safety profiles and are limited to a few agents, with very limited numbers of clinical trials for new drugs in these vulnerable patient populations. Non-invasive nutritional optimisation, stress reduction, microbiome-targeted therapies (e.g. prebiotic or probiotic supplementation) are potential options without adverse effects^331^, which should be specifically investigated in terms of their protective effect on neurodevelopmental outcomes. Postnatally, further non-invasive and safe interventions such as exposure to enriched environments, supportive parent training, and behavioural therapies, may also be considered, especially during known sensitive periods of neurodevelopment and when children are considered at greater risk due to high prenatal and/or preconceptional risk load. Another potential route for novel prevention strategies may be via the regulation of environmental pollutants, if they are found to negatively impact brain development and neuropsychiatric risk, although this may no longer be feasible for some ubiquitous agents (e.g. microplastics).

In the future, more targeted and personalised somatic manipulations of the genome (non-germline only), epigenome, transcriptome, proteome and metabolome may be feasible with biotechnological advances, if we understand enough about the regulatory mechanisms at hand in the DOMHaD framework. To achieve this, high-quality research will be necessary (Box 3), especially from large-scale and longitudinal cohort studies, to begin to unravel the time-dependent and multi-omic interacting mechanisms.

Mental well being is arguably one of the most important aspects of health to focus on, if we want to have an optimally healthy, productive and happy population. Without discounting the impact of other diseases on the length and quality of life of individuals, the perceived distinction between mental and physical health, which still persists today, has done humanity a massive disservice. Linking the manifestation of neuropsychiatric disorders to specific aberrant processes in brain formation, as proposed by the DOMHaD approach, will allow therapies to target these specifically and this approach may help substantially decrease human suffering.

## 6 Conclusions

**Figure 4:**
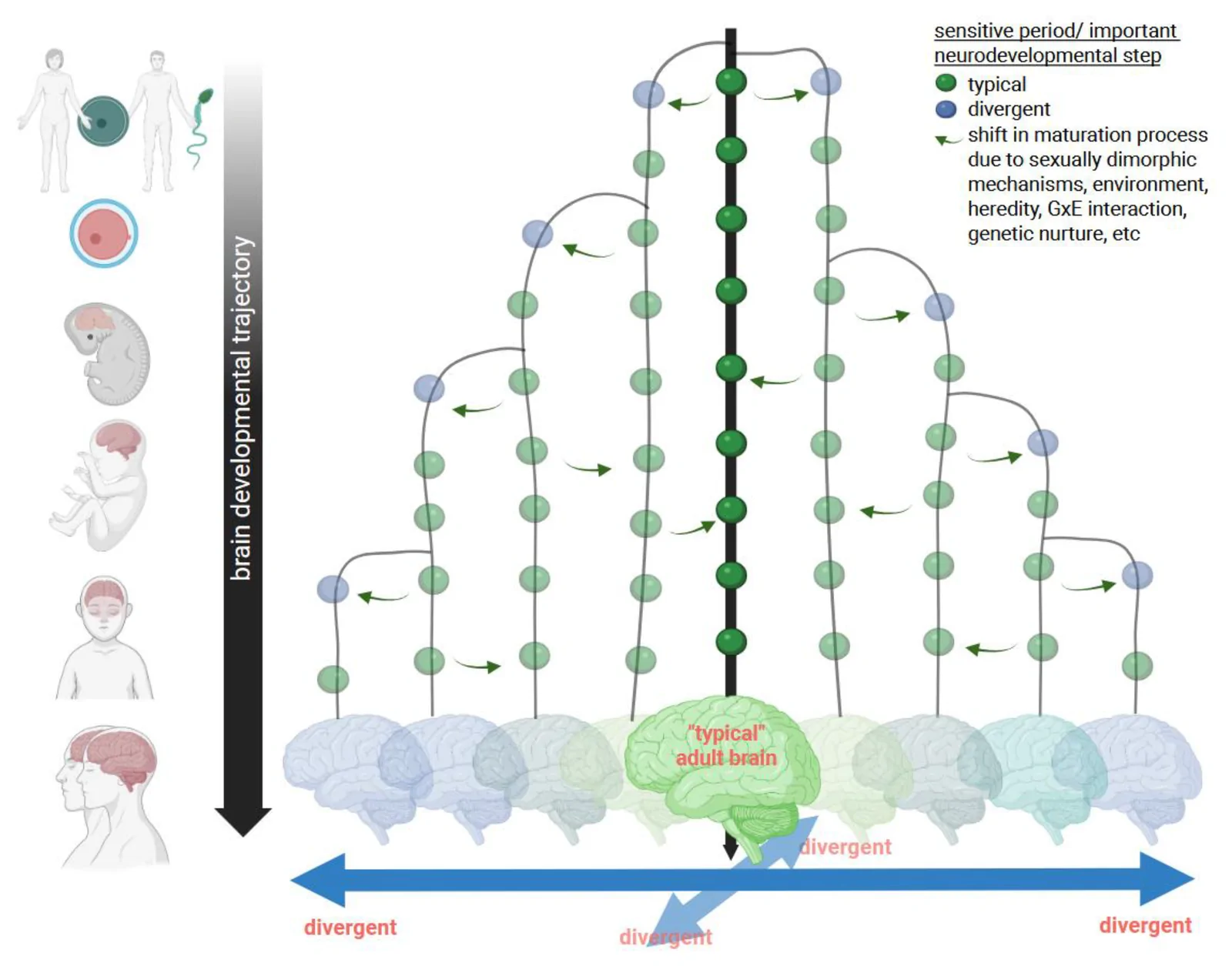
Decanalisation of normal neurodevelopmental trajectory by environmental, genetic or epigenetic modulation Under ‘typical’ circumstances, i.e. in the entirely theoretical situation where the individual matures in an environment without unexpected or unusual stimuli (or situations), with genetic and epigenetic backgrounds that do not substantially deviate from that of the broader population, brain development will follow a trajectory that culminates in the formation of a theoretical “typical” brain. These processes may be buffered and require a significant disruption to be deviated, a process termed “canalisation^”171.^ Where environmental, genetic, epigenetic signals deviate substantially, this trajectory can be altered at crucial steps in brain development, potentially resulting in an divergent adult brain to varying degrees. Importantly, genetic, epigenetic or environmental factors can likely also improve neurodevelopmental and therefore psychopathological outcomes, particularly during sensitive periods. Elucidating these interactions throughout neurodevelopment may allow us to develop new early treatments for neuropsychiatric disorders.

While the DOMHaD view of neuropsychiatric disorders and their etiologies builds on the existing theory behind DOHaD^1^, the focus of this newly proposed theoretical framework lies solely on mental health. In response to the increasing burden on societies posed by neuropsychiatric and neurological disorders, we propose an extension to the concept, in the form of a more targeted theory, focussing primarily on the brain and how changes at various timepoints and biological levels before, during and after its inception in fetal development can significantly impact outcomes relevant to psychopathology later in life (Figure 4).

A long-term view of brain development, which includes relevant parental preconceptional exposures as well as plastic brain maturation processes in the adult brain, is necessary to encompass all environmental effects on the genetic and epigenetic landscape of parents and their offspring relevant to neuropsychiatric disorders. This speaks to the complexity of this class of disorders, but also to the labile nature of the brain, which is more pronounced during specific critical and sensitive periods during the lifespan. In the context of such complexity, it can be tempting to seek the isolation of specific impacts in order to study the underlying mechanisms, however we argue that a holistic view is also necessary to be able to gain a detailed understanding of all processes leading up to the formation and maturation of a healthy brain. Interactions between different biological levels (genome, transcriptome, proteome, metabolome, cell, circuit, system, etc.) and different environmental and genetic impacts present additional layers of complexity (e.g. microbiota *x* early-life stress^333,334^) and it will be imperative to study these, as well as individual effects, to fully comprehend pathophysiological processes leading to the manifestation of psychiatric symptoms. Further complicating matters, the timing and intensity of environmental impacts often plays a central role in determining the severity of neuropsychiatric outcomes, and accumulating evidence is highlighting the role of parental (and grand-parental) environmental influences on offspring mental health.

Considering this extreme complexity, probing the causal roles and timing of specific factors in neurodevelopment will be time-consuming, expensive, and in some cases beyond current methodological reach. It may take decades and significant methodological or technological advances before we achieve a sophisticated understanding of these mechanisms and their interactions. Nevertheless, we hope to draw attention to this important field and help catalyse further research, and therefore propose the DOMHaD conceptual framework to contribute to a better understanding of the developmental origins of mental health, and facilitate novel approaches to prevention and treatment of neuropsychiatric disorders.

## Box 1 In utero environmental exposures linked to neuropsychiatric disorders: Focus on maternal immune activation (MIA) and maternal stress (MS)

### MIA

The link between neuropsychiatric disorders and maternal pathogenic infection is supported by a large number of epidemiological studies, many conducted in the aftermath of pandemics^142,146,167,168^. This led to the generation of animal models of MIA^169^. While most pathogens cannot reach the fetal compartment during infection, cytokines produced as part of the MIA response to a pathogenic infection, may directly or indirectly impact neurodevelopmental processes^170,171^. Effects may occur via functional changes in the placenta in response to MIA, altering nutritional support to the fetus, or inducing placental cytokine production^172–174^. Dysregulation of cytokines has also been observed in the fetal brain, including interleukins 1 beta, 6, 17, 13, monocyte chemoattractant protein-1 and macrophage inflammatory protein alpha^170,171,175–177^. Brain cytokine levels throughout development are tightly controlled in a time- and subregion-specific manner, and a subset of cytokines function not only as inflammatory mediators, but also as important signalling molecules in cell proliferation and neurotrophic support^178–180^. Some cytokine dysregulations in the offspring brain after MIA appear to be long-lasting^179^, and this may impact neural activity, synaptic function as well as microglia activity in addition to the effects on neurodevelopment (reviewed in^181–183^). Nevertheless, it has proven difficult to confirm that maternal cytokine responses to pathogenic infection during gestation are mediating neurodevelopmental, neurobehavioural or neuropsychiatric outcomes in human studies. While several studies support this link^184–189^, others have reported negative results^190–192^. A recent systematic review also failed to establish a link between maternal cytokine responses and brain structural changes in offspring, though the authors found that vertically transmitted viruses (such as Zika, HIV) had a significant effect^193^. The recent pandemic caused by the SARS-CoV-2 virus has led to a large-scale natural experiment to investigate the effects of viral MIA on neurodevelopmental outcomes. The results have been mixed, as some studies reported significant deleterious effects^194–197^ while others found no effect^198–200^. Interestingly, a difference between prepandemic and pandemic cohorts, but no specific association with maternal SARS-CoV-2 infection has been revealed by others^201,202^, highlighting the potential influence of non-MIA factors during the pandemic, such as social isolation during lockdowns. It should also be noted that most studies focus on mild infections, or did not stratify by maternal illness severity. Offspring neurobehavioural outcomes in children of mothers who were severely ill with SARS-CoV-2 infection show differential DNA methylation in regions relevant to neurodevelopmental processes and autism, and lower neurodevelopmental questionnaire scores at 12 months^203^. Overall, the field would greatly benefit from more large-scale longitudinal studies and the harmonisation of methodologies, focussing on combined cytokine scores rather than individual cytokine levels, specific gestational timepoints, and the systematic inclusion of specific covariates (e.g. maternal age, birth weight) in the statistical analyses^204^.

### MS

During chronic or extreme stress, levels of the glucocorticoid hormone cortisol (among others) rises in the maternal circulation. The fetal HPA axis is established in the second trimester of gestation, however fetal glucocorticoids are not produced until the third trimester when there is a physiological increase in maternal and placental glucocorticoid release^205–207^. Therefore, any earlier elevation of glucocorticoid levels in the fetal compartment has the potential to seriously disrupt offspring HPA axis maturation, and this can have long-lasting effects on the sensitivity of the HPA axis, leading to altered stress responses later in life. Due to the constitutive activity of the placental enzyme 11β-hydroxysteroid dehydrogenase (11β-HSD2), which converts maternal cortisol into the inactive cortisone, the entry of cortisol to the fetal compartment is limited to very low levels, protecting the fetal brain from direct exposure^208^.

Yet prenatal stress has been consistently linked to brain developmental abnormalities, long-term cognitive and behavioural impairments, and neuropsychiatric traits in offspring (reviewed in^209^). It was previously thought that intense or long-lasting stress may overwhelm the activity of 11β-HSD2 and let increased amounts of maternal cortisol through to the fetus^210,211^. However, work in *ex vivo* human placenta tissue has demonstrated that even with very high cortisol concentrations, and when blocking 11β-HSD2, >90 % of the cortisol did not reach the fetal circulation, suggesting robust placental mechanisms to limit cortisol transfer^208^. On the other hand, maternal glucocorticoids are also involved in trophic support of the placenta^212,213^ and in determining the timing of birth^210^, thus may be able to affect fetal brain development and subsequent mental health via processes independent of fetal HPA axis dysregulation.

## Box 2 Sexual dimorphism in DOMHaD

When considering the developmental origins of mental health and disease, a striking but consistent observation is that neurodevelopmental disorders such as ASD and SCZ are more frequent in males, while adult-onset psychiatric disorders tend to occur more frequently in females^312^. It remains difficult to definitively exclude the possibility of systematic and widespread underdiagnosis of these types of neurodevelopmental disorders in females, due to the historical predominance of male-focussed investigations in neuropsychiatric research. Regrettably, this bias appears to have additionally led to a lack of understanding of the symptom complexes experienced specifically by girls, women and non-cisgender identities suffering from particular disorders^313^. In addition, diagnostic frameworks used in psychiatric diagnoses, such as the Diagnostic and Statistical Manual of Mental Disorders 5 (DSM-5)^314^, may exacerbate disparities through the use of gender-normative criteria that fail to account for diverse gender identities. For example, autism spectrum disorder (ASD) is often underdiagnosed in nonbinary and transgender individuals because DSM-5 criteria and diagnostic tools are normed on cisgender male social behaviours and developmental trajectories^315^. This is despite evidence that this population actually has higher rates of neurodevelopmental and other psychiatric disorders^316^. A gendered diagnostic lens can result in missed or delayed diagnoses, particularly for those whose neurodevelopmental presentations do not align with stereotypically “male” diagnostic criteria. Such structural bias highlights the need to critically evaluate how cisnormative assumptions in diagnostic manuals shape psychiatric visibility and access to care^317^. Beyond these diagnostic biases, recent evidence suggests that sexual dimorphisms in neurodevelopmental disorders are in fact a result of differential regulation of developmental processes at several levels. The concept of ‘male vulnerability’ is often used in this context^318^, as male-specific mechanisms appear more labile and prone to disturbances by environmental factors. Interestingly, the phenomenon of increased male vulnerability may extend to intergenerational and transgenerational inheritance, where male offspring generally appear more severely impacted by environmental impacts on the parental and grand-parental (etc.) generations^14,319–322^. However, it is still not entirely clear whether this is a robust finding or biased by the choice of behavioural tests (which may inherently be more sensitive to male behavioural alterations) or similar factors, and this question requires further investigation. Genetic determination of sex depends on the presence of either XX or XY sex chromosomes in the zygote. In particular, the sex-determining region of the Y chromosome is thought to be essential in sex specification during embryonic development^323^, and it has been considered essential to steer development away from the “default” female condition. On the other hand, there are additional potentially significant differences that arise from the XX vs XY distinction between females and males: In males, all somatic cells have one X and one Y chromosome, leading to relatively homogenous cell populations, at the genetic and transcriptional level, within a given tissue in males^323^. On the other hand, in females, the existence of two X chromosomes in all somatic cells leads to the need for X-inactivation of either the maternally inherited X or the paternally inherited X chromosome. This occurs randomly in each somatic cell, thus leading to the existence of two genetically distinct cell populations within each tissue, where some cells have the paternal X chromosome inactivated while others have the maternal X chromosome inactivated. Gene expression in female tissues therefore follows a mosaic pattern and the overall tissue gene expression is the average of the expression across the two cell subpopulations. It has been suggested that this mosaicism may lead to higher variability in certain phenotypes, including cognition^323^. In addition, when the emergence of neurodevelopmental disturbances is based on aberrations in gene expression arising from an X chromosome, the presence of functionally distinct cell populations provides a protective effect where not all cells will be equally affected. Similarly, one might speculate that in response to a given environmental perturbation, one of these cell populations may be more resilient to insults than the other – leading to an overall milder impact on the neurodevelopmental trajectory in females.

The importance of genetics in controlling neurodevelopment in a sexually dimorphic manner has also been highlighted by several studies where differential gene expression was observed between developing male and female mouse brains, at stages where the gonads are not yet developed, thus prior to the start of sex hormone production^324,325^.

Later in gestation, sex hormones do however play a pivotal role in modulating brain developmental processes. In males, the differentiated testes secrete androgens in a tightly orchestrated manner, with multiple peaks in hormone production around birth, a mechanism that was first elucidated in rats^326,327^. More recently, the action of these hormones has been linked to the inhibition of cell-death mechanisms in some brain areas in males, and increased cell death in other brain areas, when compared to females^328^. Interestingly, these differences in cell numbers in brain areas between the sexes are present in adulthood^329^, highlighting the long-term impact of the sex-specific hormonal control of neurodevelopmental processes on brain structure and, by extension, brain function.

While there have not been any direct examinations of male and female neural developmental differences amand their relationship with psychiatric diagnoses later in life, the evidence discussed above suggests that, at several stages, distinct mechanisms may be at play in males and females. Future studies should therefore investigate the role of neurodevelopment in the etiology of psychiatric disorders in a sex-specific manner, which would likely reveal different underlying mechanisms.

## Box 3 Proposed strategies for research within the DOMHaD framework

While the DOMHaD theoretical framework is supported by compelling evidence, the complexity of the interactions between brain development, the environment, genetics, epigenetics, and other biological factors, mean that there are still many unanswered questions. To fully leverage DOMHaD in the improvement of mental health treatment and prevention, we need to understand the molecular mechanisms underlying these interactions, and their impacts on brain development, behaviour and mental health. Research should also investigate whether (and under which conditions) deviations from normal brain development under control of specific environmental, genetic and epigenetic influences can be reversed or mitigated, so that we may move towards evidence-based therapeutic strategies targeting specific sensitive timepoints and neurodevelopmental processes, e.g. via “prescribed” extra nurture and care protocols in early infancy after early neglect experiences or in genetically predisposed individuals. This endeavour will require both human and animal research, which in concert will allow the investigation of disorder-specific neurodevelopmental mechanisms and their targeted treatment, as well as the detailed interrogation of molecular mechanisms and their causal contributions, respectively. We propose several important considerations for future research to ensure the generation of high-quality evidence that may be used to enrich the DOMHaD framework and ultimately improve outcomes for patients across neuropsychiatric disorders, including those not traditionally associated with neurodevelopment.

HUMAN RESEARCH

- Collect longitudinal data to explore specific neurodevelopmental windows in the same individual, This may require the adoption of strategies for participant retention, such as maintaining contact or providing incentives^332^.
- Ensure adequately powered sample sizes, ideally with large prospective birth cohort studies.
- Expand inclusion of individuals and controls in studies (family designs, e.g. parent-individual trios, dyads).
- Expand data types collected from the same individuals (multi-omic analyses) to allow for the investigation of relationships between different biological levels in the context of DOMHaD.
- Use transdiagnostic markers, psychiatric endophenotypes and expand neurodevelopmental studies to neuropsychiatric disorders beyond SCZ, ASD and ADHD.
- Where ethical and feasible, leverage natural experiments (e.g. COVID-19 pandemic).

ANIMAL MODEL RESEARCH

- Focus on exploring causal mechanisms between associations coming directly from human research to stay highly translational.
- Novel interventions should be first tested in animals, but should have rapid translation in mind.
- High-quality animal models should be used, with specific considerations for construct and face validity in terms of neurodevelopmental changes observed in specific disorders. Predictive validity, where possible, is a further consideration. Alternatively, a focus on specific disruptions (via particular environmental impacts or genetic modifications), their causal involvement in determining maladaptive behaviour, and the potential rescue of resulting deficits may have transdiagnostic value.
